# Network Candidate Genes in Breeding for Drought Tolerant Crops

**DOI:** 10.3390/ijms160716378

**Published:** 2015-07-17

**Authors:** Christoph Tim Krannich, Lisa Maletzki, Christina Kurowsky, Renate Horn

**Affiliations:** Institute of Biological Sciences, Department of Plant Genetics, University of Rostock, Albert-Einstein-Str. 3, 18059 Rostock, Germany; E-Mails: christoph.krannich@uni-rostock.de (C.T.K.); lisa.maletzki@gmx.de (L.M.); christina.kurowsky@uni-rostock.de (C.K.)

**Keywords:** candidate genes, breeding, yield penalty, drought tolerance, genetic variants

## Abstract

Climate change leading to increased periods of low water availability as well as increasing demands for food in the coming years makes breeding for drought tolerant crops a high priority. Plants have developed diverse strategies and mechanisms to survive drought stress. However, most of these represent drought escape or avoidance strategies like early flowering or low stomatal conductance that are not applicable in breeding for crops with high yields under drought conditions. Even though a great deal of research is ongoing, especially in cereals, in this regard, not all mechanisms involved in drought tolerance are yet understood. The identification of candidate genes for drought tolerance that have a high potential to be used for breeding drought tolerant crops represents a challenge. Breeding for drought tolerant crops has to focus on acceptable yields under water-limited conditions and not on survival. However, as more and more knowledge about the complex networks and the cross talk during drought is available, more options are revealed. In addition, it has to be considered that conditioning a crop for drought tolerance might require the production of metabolites and might cost the plants energy and resources that cannot be used in terms of yield. Recent research indicates that yield penalty exists and efficient breeding for drought tolerant crops with acceptable yields under well-watered and drought conditions might require uncoupling yield penalty from drought tolerance.

## 1. Introduction

Producing enough food for the growing world population will be a challenge in the next 50 years [1]. Regarding abiotic stress factors, drought is probably the environmental factor that has worldwide the strongest negative impact on crop productivity [2]. Several factors will worsen the situation in the coming years [3]: (1) climate change will lead to differences in rain patterns all over the world with reduced rainfalls and more extensive drought periods, especially in subtropical areas; (2) continuous loss of arable land by soil erosion, urbanization and salinization of irrigated fields will reduce land available for agriculture; and (3) crop production, especially in the developing countries, is more and more relocated into more drought-prone areas with poor soil qualities. Even relatively mild droughts, such as experienced in the UK, can reduce yields obtained on rain-fed agriculture compared to maximal yields (under irrigated conditions or in years with more rain) by as much as 30%–60% [4].

Agricultural drought addresses the situation in which the water available to the plants through rainfall and/or irrigation is insufficient to meet the transpiration needs of the crop, which leads to yield reductions [5]. The crop yield gap (Yg) is defined as the difference between the yield potential Yp (irrigated systems) or water-limited yield potential Yw (rainfed) and the average actual farm yield (Ya) [6]. The agricultural drought will differ between crops because of two major factors (crop water demand and crop water supply) [7].

Drought tolerance is a very complex trait as it depends not only on the severity of the drought (mild or severe), but also on the developmental stage of the plant as well as the duration [8,9]. From the physiological point of view, survival (and/or recovery) is the major aim in plant stress tolerance, whereas from the agricultural point of view crop yield is the trait that determines a drought tolerant crop [10]. Different types of drought tolerance can be present within a crop like in wheat (*Triticum aestivum*). The genotype RAC875 and Excalibur demonstrated different strategies for drought tolerance under the same drought scenario [11]. RAC875 showed a more conservative strategy by producing fewer tillers, higher number of grains per tiller and by revealing a moderate osmotic adjustment. RAC875 plants stored more water-soluble carbohydrates and the leaves were more waxed and thicker, both under well-water and drought conditions. On the other hand, Excalibur showed a more responsive strategy by higher osmotic adjustment, low abscisic acid (ABA), high stomatal conductance and rapid recovery after stress. Leaves rolled under dry conditions, higher number of tillers were produced and aborted under water-limitations, but the loss was compensated by the production of more grains per tiller after recovery. Both strategies for drought tolerance would be interesting from the breeding perspective, especially as these two types may perform differently under other drought scenarios.

Plant hormones are involved in every aspect of plant development as well as in the reaction of plants to abiotic and biotic stress [12,13]. The overlap in hormone-regulated pathways and interactions indicate a complex network of extensive cross talk between the different hormone signaling pathways during adaptation to drought [12,14]. Downstream of the signaling pathways more than 50 families of transcription factors (TFs), e.g., ABA response element binding factors (AREBs), basic region/leucine zipper (bZIP) TFs, drought responsive element binding proteins (DREBs), ethylene response factors (ERFs), MYC/myeloblastosis (MYB) TFs, and no apical meristem/ATAF/CUP-shaped cotyledons (NACs) function as integrators of the complex network of drought regulated genes [15,16,17].

For the proof-of-concept as well as functional analysis, transgenic approaches have been successfully used for a better understanding of the networks underlying drought tolerance and for identifying candidate genes for breeding drought tolerant crops. However, in most cases only the survival (and/or recovery) under drought has been addressed, but not the yield aspect. Our knowledge about drought tolerance now needs to be applied to produce drought tolerant crops with improved yields (and yield stability).

In this review, we will highlight some candidate genes out of the complex network engaged in drought tolerance, which involves biosynthesis of plant hormones and their signaling pathways, osmoprotective strategies as well as the detoxification of reactive oxygen species (ROS), that seem to be most promising to be analyzed with regard to the genetic variation in drought tolerant and drought sensitive plant material.

## 2. Role of Plant Hormones in Drought Response

### 2.1. Abscisic Acid (ABA) Biosynthesis, Catabolism and Signaling Pathways Are Major Players under Drought Conditions

Abscisic acid (ABA) plays an important role in adaptive responses to environmental stresses such as drought, but also in developmental processes such as seed maturation, dormancy and senescence [18,19]. Genes involved in ABA biosynthesis, catabolism and signaling network represent interesting candidate genes for breeding crops improved in drought tolerance.

ABA synthesis starts in the plastids with zeaxanthin, which is a product of the 2-*C*-methyl-D-erythritol-4-phosphate (MEP) pathway [20]. The enzyme zeaxanthin epoxidase (ZEP) converts the C_40_ carotenoid zeaxanthin via the intermediate antheraxanthin to all-trans-violaxanthin [18]. The reverse reaction is catalyzed by the violaxanthin de-epoxidase (VDE). This so-called xanthophyll cycle is supposed to be relevant for the protection of photosystems from photooxidative damage under intense light conditions [21,22]. In the following, all-trans-violaxanthin can be transformed to either 9-*cis*-violaxanthin or 9′-violaxanthin by a yet unknown enzyme. The rate-limiting step of ABA biosynthesis is the formation of xanthoxin via oxidative cleavage from either of these precursors by the enzyme 9-*cis*-epoxycarotenoid dioxygenase (NCED). Overexpression of NCED leads to higher levels of ABA, a reduction of the transpiration rate in leaves and thus enhances drought tolerance [23,24]. But also overexpression of *ZEP* in *Arabidopsis thaliana* conferred greater tolerance indicating that this enzyme might be limiting with regard to some stress response [25]. Xanthoxin is transported into the cytosol were it is converted to abscisic aldehyde by one of the short chain dehydrogenase/reductase (SDR) family, e.g., abscisic acid 2 (*ABA2*) in *Arabidopsis* [26]. The last step in the synthesis of ABA is catalyzed by abscisic aldehyde oxidase (AAO). This AAO requires a sulphurylated form of a molybdenum cofactor (MoCo) for its activity [27,28]. The MoCo sulfurase is encoded by low expression of osmotically responsive genes (*LOS5*)/abscisic acid 3 (*ABA3*) gene [27,29]. Overexpression of the molybdenum cofactor sulfurase gene enhanced drought tolerance in soybean [30] by increasing ABA accumulation, reducing water loss through reduced opening of the stomata and increased activities of superoxide dismutase (SOD), peroxidase (POD) and catalase (CAT). This overexpression resulted in considerably higher yields in form of more shoot and root biomass as well as more pods under water limited conditions. Yields were comparable to the wild type under well-watered conditions. Also for rice (*Oryza sativa*) [31] and for maize (*Zea mays*) [32], enhanced drought tolerance combined with improved yield under drought stress was observed in transgenic plants overexpressing *LOS5*/*ABA3*. Two catabolic pathways, hydroxylation predominantly by ABA 8′-hydroxylase and conjugation by ADP glucosyltransferase, are known for ABA [33,34]. ABA levels are maintained through the balance between *de novo* biosynthesis and catabolism, rather than only biosynthesis [35]. Drought leads to upregulation of *CYP707A* genes encoding for ABA 8′-hydroxylases, which is further increased upon rehydration. The catabolic products like ABA glucosyl ester and phaseic acid are stored in the vacuole or the apoplast pool. Under drought conditions, the enzyme β-glucosidase can cleave the conjugated ABA and release ABA, which can then be transported to the guard cell to induce stomatal closure [36].

The ABA signal transduction pathway has been well analyzed using *A. thaliana* as model plant [20,37,38]. The signaling pathway comprises three major components: a group of ABA receptors in the cytosol that binds to protein phosphatase 2C (PP2C) in the presence of ABA and (sucrose non-fermenting) SNF1-related protein kinase 2 (SnRK2). The ABA receptors represent a complex of pyrabactin resistance (PYR), PYR-like (PYL) and regulatory component of ABA receptor (RCAR). All components of the complex are encoded by multigene families, which results in redundancy in function [19,39]. Variation in one of the genes might not exhibit a great effect and thereby reduces the chance to use variation in these genes to breed for drought tolerance. Quadruple mutants had to be applied to demonstrate the role of the receptors. In the presence of ABA, PP2C is bound by the ABA receptor PYR/PYL/RCAR, which frees SnRK2 to be phosphorylated. PP2C like ABA insensitive 1 (ABI1), ABI2 or other similar ones belong to group A of the protein phosphatase gene family [40]. SnRKs are serine/threonine protein kinases, which are divided in three groups: SnRK1, SnRK2 and SnRK3 [41]. The SnRK2 family represents the key regulator of plant response to abiotic stress. Despite the multigene families involved, overexpression of a single *PP2C* gene, e.g., *OsPP108* from rice, leads to low sensitivity to ABA and high drought tolerance in *Arabidopsis* [42]. Also constitutive overexpression of PYL5 in rice enhanced drought tolerance [43]. However, this was accompanied with deleterious effects on total seed yield. Overexpression of PYL1/RCAR, PYL5/RCAR8 or PYL8/RCAR3 gave more tolerance to drought in *Arabidopsis* [20]. Table 1 summarizes the potential candidate genes from the ABA biosynthesis, catabolism and signaling pathway that might be evaluated in breeding for drought tolerant plants.

ABA plays the key role in plant adaptation to water-limited conditions. The fastest reaction of the plants under drought conditions is an increase in ABA level, which triggers the expression of ABA-responsive genes and induces stomatal closure [12,44]. As a consequence water loss via transpiration is reduced. Overexpression of enzymes of the biosynthesis pathway, e.g., ZEP or NCED leads to an increased ABA level [23,25], which improves drought tolerance by reducing transpiration. Growth regulation under water-limited conditions is also primarily mediated by ABA and ethylene [14,45]. In addition, XERICO (Greek for “drought tolerant”), a RING (really interesting new gene)-H2 zinc transcription factor, functions as a node between abiotic stress response and development by linking gibberellic acid (GA) and the ABA signaling pathway [46,47]. The effect of different ABA levels on yields under well-watered and drought conditions have still to be studied [14]. In addition, a better understanding of the cross talk with other plant hormone signaling pathways might offer the chance to improve breeding for drought tolerant crops.

**Table 1:** Candidate genes of the abscisic acid (ABA) biosynthesis, catabolism and signaling.

Gene	Gene Function	Abiotic Stress Tolerance	Reference
Zeaxanthin epoxidase (*ZEP*)	ABA biosynthesis	Improved tolerance to drought stress and salt	[22,25]
9- *cis*-Epoxycarotenoid dioxygenase (*NCED*)	ABA biosynthesis	Improved drought tolerance by overexpression in tomato	[23,48]
Molybdenum cofactor sulfurase (*LOS5*/*ABA3*)	ABA biosynthesis	Increased drought tolerance in maize and soybean combined with higher yields	[30,32]
ABA 8′-hydroxylase	ABA catabolism	Upregulated during drought stress and rehydration: reduction in ABA level	[35]
Abscisic acid receptor (*PYL*)	ABA signaling network	Enhanced drought and salinity tolerance	[19,43]
Protein phosphatase 2C (*PP2C*)	ABA signaling network	Induced by drought, repressed by oxidative stress and heat shock	[49,50]
Serine/threonine protein kinase (*SnRK2*)	ABA signaling network	Enhanced tolerance to drought, salt, and freezing stress	[51,52]
SCARECROW (*SCR*)	Transcription factor, structural root differentiation	Binding to regulatory regions of stress-responsive genes; Regulating abscisic acid responses in *Arabidopsis*	[51]

### 2.2. Role of Ethylene Biosynthesis, Catabolism and Signaling Pathway for Drought Tolerance

The gaseous plant hormone ethylene has been reported to be involved in a large variety of developmental processes, including seed germination and ripening, as well as abiotic or biotic stress responses, e.g., stomatal closure. Both ethylene biosynthesis and signaling are important components of plant responses to different abiotic stresses such as drought and salt. As there are numerous genes involved in ethylene synthesis and signaling, it is important to identify candidate genes, that are significantly correlated to stress responses and therefore useful for breeding drought tolerant crops (Table 2).

**Table 2:** Candidate genes for ethylene synthesis and signaling.

Gene	Gene Function	Abiotic Stress Tolerance	Reference
1-Aminocyclopropane-1-carboxylic acid oxidase (*ACO*)	Ethylene biosynthesis	Induced expression during wilting in tea plants promotes decreased metabolism and energy consumption by shedding of older leaves	[53]
1-Aminocyclopropane-1-carboxylic acid synthase (*ACS*)	Ethylene biosynthesis	Delayed senescence in absence of *ACS* in maize	[54]
1-Aminocyclopropane-1-carboxylic acid synthase 7 (*ACS7*)	Ethylene biosynthesis	*ACS7*-knock-out promotes tolerance to salt, osmotic and heat stresses	[55]
Ethylene-overproducer 1 (*ETO1*)	Limitation of ACS in ethylene biosynthesis	*ETO1*-deficient mutants in *Arabidopsis* exhibited delayed stomatal closure in response to drought	[56]
Ethylene overproducer 1-like (*ETOL1*)	Ethylene biosynthesis and energy metabolism	*ETOL1*-overexpressing rice more tolerant to drought and submergence	[57]
Ethylene response 1 (*ETR1*)	Ethylene signaling	Cross-link between ethylene and H_2_O_2_ signal transduction in *Arabidopsis*, stomatal closure; inhibition of seed germination under salt stress	[58,59]
Ethylene response 2 (*ETR2*)	Ethylene signaling	*ETR2* promotes seed germination under NaCl stress in *Arabidopsis*	[59]
Ethylene insensitive 2 (*EIN2*)	Ethylene signaling	*EIN2* downregulated in *Arabidopsis* during salt and osmotic stress, *EIN2*-deficient mutants sensitive to salt and osmotic stress	[60]
Ethylene insensitive 3 (*EIN3*)	Downstream element of ethylene signaling pathway	*EIN3*-deficient mutants in *Arabidopsis* exhibited oxidative stress after exposure to salt stress	[61]
Ethylene response factor 1 (*ERF1*)	Jasmonate and ethylene signaling	*ERF1*-overexpressing *Arabidopsis* more tolerant to drought and salt stress	[62]
Ethylene responsive element binding protein 1 (*EREBP*)	Connection between different stress signal transduction pathways	Expression induced in oil palm fruits in response to abiotic stress (drought, cold and salinity)	[63]
Ethylene response factor 5 (*ERF5*)	Vegetative growth and plant development	*ERF5*-overexpressing tomatoes more tolerant to drought and salt stress	[64]
Ethylene response factor 6 (*ERF6*)	Cell proliferation and leaf growth	*ERF5/ERF6*-deficient *Arabidopsis* mutants less affected by stress	[65]
Jasmonate and ethylene response factor 1 (*JERF1*)	Activation of stress responsive genes, proline synthesis and ABA biosynthesis key enzymes	*JERF1*-overexpressing rice more tolerant to drought stress	[66]

Two main groups of enzymes, comprising 1-aminocyclopropane 1-carboxylic acid (ACC) synthases (ACSs) and ACC oxidases (ACOs) are indispensable for ethylene biosynthesis. While ACSs are responsible for converting *S*-adenosyl methionine (SAM) to ACC, the family of ACOs catalyzes the conversion of ACC to ethylene [67]. The conversion of SAM to ACC represents the rate-limiting step that is catalyzed by different isoforms of ACS. In addition, 1-malonyl aminocyclopropane-1-carboxylic acid (MACC) transferase (MACCT) is considered to be a sink for ethylene, as its activity converts ACC into the non-volatile MACC [68]. In tea (*Camellia sinensis*) plants, a gene of the ACO-group is connected with drought tolerance [53]. At the wilting stage the ACO expression increased, possibly in order to decrease energy consumption and metabolism through shedding of older leaves. In addition, the frequently observed involvement of ethylene in leaf senescence can also be assigned to another biosynthetic enzyme, in this case a member of the ACS family. In *Arabidopsis*, ACS7-deficient mutants exhibited increased tolerance to salt stress [55]. On the molecular level, ACS7 loss-of-function mutants had a higher expression of several salt responsive genes including sodium/hydrogen exchanger 1 (*NHX1*), which encodes a tonoplast sodium/proton antiporter [69]. In ACS6-deficient maize mutants leaf senescence under normal conditions was delayed and drought-induced senescence was inhibited [54]. Furthermore it was demonstrated, that the leaves maintained physiological and biochemical function under drought stress. In fact ACS6-deficient plants even exhibited a significantly higher CO_2_ assimilation rate, which appears to be a major advantage in terms of photosynthetic activity under stress conditions as a substantial factor for crop yield.

In ethylene biosynthesis ACS is the rate-limiting enzyme and therefore is strictly regulated in order to maintain a distinct level of ethylene production. Ethylene overproducer 1 (ETO1) serves as a main component in ACS regulation through promoting the degradation of ACS by a proteasome dependent pathway [56]. ETO1-deficient mutants in *Arabidopsis* however exhibited distinct responses upon drought stress. Ethylene interferes with ABA mediated closure of stomata. Thereby mutants showed a delayed stomatal closure, resulting in a higher degree of water loss by transpiration during drought [70]. Furthermore, an ethylene overproducer 1-like (ETOL1) protein, with a homologous function to ETO1, has also been reported to benefit drought tolerance during distinct developmental stages in rice (*Oryza sativa*) [57].

The ethylene signaling pathway is more complex, due to the large number of genes that are involved and the multiple processes and responses that are regulated [39,71]. The signaling network includes the receptor complexes I (ETR1/ERS1) and II (ETR2/ERS2/EIN4), that are membrane associated and connected to a negative constitutive triple response 1 (CTR1) protein kinase. Complex I consists of ethylene response 1 (ETR1) and ethylene response sensor 1 (ERS1), while complex II has three components consisting of ETR2, ERS2 and ethylene insensitive 4 (EIN4). In absence of ethylene CTR1 negatively regulates the ethylene insensitive 2 (EIN2) protein. After binding of ethylene to one of the receptor subfamilies, a signal peptide (EIN2C) is cleaved from EIN2 and transported into the nucleus, where the ethylene insensitive 3 (EIN3)/ethylene insensitive like 1 (EIL1) dependent gene transcription is activated [71]. Different elements of this proposed ethylene signaling pathway were reported to be associated with stress responses after drought and salt stress.

ETR1 represents an important link between ethylene and H_2_O_2_ signaling, as *Arabidopsis* mutants deficient of ETR1 were insensitive to H_2_O_2_ and thereby lacking ethylene mediated stomatal closure [58]. Under salt stress conditions ETR1-deficient mutants in *Arabidopsis* germinated faster and with a shorter delay in the onset of germination [59]. In contrast, ETR2 inhibits ETR1 and stimulates seed germination under salt stress underlining the different receptor functions.

Moving downstream, the signaling pathway EIN2 facilitates the signal transduction from the cytosol into the nucleus. During salt and osmotic stress EIN2 has been reported to be downregulated in *Arabidopsis*, while EIN2-deficient mutants were highly sensitive to salt and osmotic stress and showed increased ABA levels [60]. This effect can possibly be considered as crosstalk between ethylene and ABA signaling that also integrates stress signals. After binding of ethylene to a receptor subfamily and EIN2-mediated transport of the signal into the nucleus, the downstream transcription factors such as EIN3 and other ethylene insensitive-like proteins (EILs) are activated [72].

Specifically, EIN3 is essential for ethylene signaling, as EIN3-deficient mutants exhibited severe cell growth inhibition as well as accelerated senescence. The expression of EIN3 during salt stress is induced, suggesting that EIN3 could be a key factor in salt stress signaling [61]. In addition, EIN3-deficient mutants in *Arabidopsis* exposed to salt stress also showed enhanced oxidative stress, underlining the important role in response to salt stress. Another class of ethylene dependent genes, that have received major attention, are ethylene responsive element binding factors (EREBFs), often also referred to as ethylene response factors (ERFs), which like EIN3 represent downstream components of the signaling pathway. The unique feature of this ERF family is a highly conserved DNA binding domain. Several ERFs were reported to participate in drought response in different plants [73,74].

Rice mutants overexpressing the response factor jasmonate and ethylene response factor 1 (JERF1), which encodes a tomato ERF-protein, exhibited enhanced drought response [66]. This effect is mediated by the expression of stress responsive genes, including key enzymes of proline-biosynthesis. Furthermore, also ABA-biosynthesis was activated by JERF1, which illustrates the high impact of a single ERF on diverse signaling and biosynthesis pathways. Another example of crosstalk mediated by a single ERF was observed in *Arabidopsis*. ERF1 represents a component of both jasmonate (JA) and ethylene signaling and is highly induced under drought and salt stress conditions [62]. The ERF1-overexpressing mutants showed improved tolerance to drought, salt and heat. As the stress induction was inhibited by ABA, ERF1 integrates signals of different plant hormones, resulting in multiple stress responses.

Concerning leaf growth inhibition under osmotic stress, the redundant transcription factors ERF5 and ERF6 were reported to be central components [65]. ERF5/ERF6-deficient mutants in *Arabidopsis* exhibited better growth under short and long term osmotic stress. ERF6 regulates gibberellin-biosynthesis (GA) through transcriptional induction of GA 2-oxidase 6 (GA2-OX6). Thus GA-breakdown is triggered, leading to stabilization of DELLA proteins, known to negatively influence growth. ERF5-overexpressing tomatoes (*Solanum lycopersicum*) were more tolerant to abiotic stresses including drought [64]. As the expression of ERF5 was also induced by ABA, this transcription factor could also depict another cross-link between ethylene and ABA-signaling. In oil palm (*Elaeis guineensis*) a new member of the ERF family, which appears to be closely related to several stress responses including drought, has been identified [63]. This ethylene responsive element binding protein (EREBP) was induced after the onset of different stresses including drought, salt and cold. Furthermore, increased expression has also been observed after treatment with different plant hormones including ethylene and ABA. These results suggest a possible cross-link between different stress response signaling pathways, mediated by a single transcription factor.

In summary, ethylene metabolism and signaling influence a large variety of stress responses in plants. Particularly the ethylene signaling pathway appears to be a major cross-link between ethylene and other plant hormones, e.g., ABA and GA. The reported interactions between ethylene and other plant hormones also benefit immediate stress responses such as stomatal closure as well as long term adaptations, e.g., improved growth under drought conditions. Especially candidate genes that are related to maintenance of growth under low water conditions during different developmental stages are agronomically relevant as they provide an efficient resource for crop improvement and counteract yield penalties.

## 3. Protection of the Cells against Osmolytic and Oxidative Damages

### 3.1. Osmolytes: Glycine Betaine, Proline and Trehalose

In order to protect the membrane-integrity and thereby an aqueous environment for organelles, plants possess various mechanisms to control water efflux and influx. As a part of controlling water movement, the biosynthesis of different substances that function as osmotically active solutes (osmolytes) is induced [75]. Especially during stress responses and acclimation osmolytes are essential for maintaining cellular functions as well as protein and enzyme activity. Different osmolytes in plants have been reported to be associated with drought and salt stress, including proline, glycine betaine and trehalose. Enzymes involved in the metabolism of these osmolytes represent potential candidates for breeding drought tolerant plants (Table 3).

**Table 3:** Candidate genes for osmolyte biosynthesis and cell protection.

Gene	Gene Function	Abiotic Stress Tolerance	Reference
Betaine aldehyde dehydrogenase (*BADH*)	Osmolyte biosynthesis	BADH from spinach in potato improved drought tolerance	[76]
Δ^1^-pyrroline-5-carboxylate synthetase (*P5CS*)	Osmolyte biosynthesis	Proline: most frequent osmolyte in water-stressed plants	[77]
Proline oxidase/dehydrogenase 1 (*PDH1*)	Osmolyte biosynthesis	Proline: most frequent osmolyte in water-stressed plants	[77]
Δ^1^-pyrroline-5-carboxylate reductase (*P5CR*)	Osmolyte biosynthesis	Proline: most frequent osmolyte in water-stressed plants; enhanced drought tolerance in soybean	[78]
Trehalose-phosphate synthase 1 (*TPS1*)	Osmolyte biosynthesis	Transgenic expression in potato improved drought tolerance	[79]
DNA-binding protein 4	Transcription factor	Induced by drought	[51]
Dehydration-responsive element binding factor 1 (*DREB1B*)	Transcription factor	*Arabidopsis* gene in transgenic potato improved drought tolerance	[80]

A transgenic potato (*Solanum tuberosum*) cultivar, containing a betaine aldehyde dehydrogenase (*BADH*) gene from spinach (*Spinacia oleracea*) under the control of a stress induced *Arabidopsis* promoter, has been reported to exhibit improved growth after induction of BADH by NaCl and drought stress [76].

Another highly relevant osmolyte in plants is the aforementioned proline, which accumulates under different stress conditions [81]. The role of the amino acid proline in plant cells is considered to be diverse and includes contributing to osmotic adjustment and stabilization of sub-cellular structures [82]. The enzyme Δ^1^-pyrroline-5-carboxylate synthetase (P5CS1) is a major component in proline biosynthesis. A recent study with P5CS1-deficient *Arabidopsis* mutants indicated that proline synthesis is required in order to maintain growth at low water availability [77]. In addition, the study also confirmed the necessity of proline-catabolism, as proline dehydrogenase 1 (PDH1)-deficient *Arabidopsis* mutants, with blocked proline catabolism, exhibited decreased root growth, fresh weight and dry weight. Furthermore additional components of the proline biosynthetic pathway have also been reported to be associated with stress responses. In transgenic soybean (*Glycine max*) with a Δ^1^-pyrroline-5-carboxylate reductase (*P5CR*) gene and the antisense construct from *Arabidopsis*, it was found that proline might enhance survival during drought stress [78]. The *P5CR* gene and antisense construct were manipulated using an inducible heat shock promoter (IHSP). In contrast to wild-type (WT) plants, transformed plants failed to survive drought stress at 37 °C, suggesting proline as an essential component of tolerance to drought.

Two transgenic potato lines, which expressed a trehalose-6-phosphate synthase (*TPS1*) gene from yeast (*Saccharomyces cerevisiae*) were found to be more effective in keeping water and acceptable levels of photosynthesis during drought compared to WT-plants [79]. However under optimal growth conditions the transgenic lines were less efficient in terms of CO_2_ fixation than control plants, as the stomatal densities were reduced.

### 3.2. Aquaporins

A subfamily of major intrinsic proteins (MIPs), referred to as aquaporins (AQPs), function as intercellular water channels and thereby enable rapid water and solute transport between cells (Table 4). Since the first AQP was discovered more than 20 years ago [83], a large variety of AQPs in different plants and tissues were reported. The family of AQPs can further be sub-classified into plasma membrane intrinsic proteins (PIPs), tonoplast intrinsic proteins (TIPs), nodulin-26 like intrinsic proteins (NIPs), small basic intrinsic proteins (SIPs) and x intrinsic proteins (XIPs) [84]. During water limiting conditions, with low water availability and increased transpiration, an effective water transport across membranes is indispensable for plants in order to maintain growth and favourable metabolic levels in an unfavourable environment.

In rice (*Oryza sativa*) plants grown in the presence of NH_4_, expression levels of AQPs were elevated in roots, resulting in an increased root protoplast water permeability and root hydraulic conductivity [85]. Based on these findings root water uptake and drought resistance could be improved by efficient nitrogen supply.

**Table 4:** Candidate genes for aquaporins.

Gene	Gene Function	Abiotic Stress Tolerance	Reference
*PIP2;1*	Water transport	*PIP2;1* transcription downregulated in *Arabidopsis*; *PIP2;1* degradation benefits drought tolerance	[86,87]
*GoPIP1*	Water transport	*GoPIP1* overexpressing *Arabidopsis* mutants more sensitive to drought	[88]
*NtPIP1;1*, *NtPIP12;1*	Water transport	Downregulation of *NtPIP1;1* and *NtPIP2;1* in tobacco after drought stress	[89]
*NtAQP1*	Water transport	*NtAQP1* transcription upregulated in tobacco after drought stress	[89]
*MaPIP1;1*	Water transport	*MaPIP1;1* transcription in banana upregulated during drought stress; *MaPIP1;1* overexpressing *Arabidopsis* mutants more tolerant to drought	[90]

Furthermore, *Arabidopsis* mutants overexpressing a RING membrane-anchor E3 ubiquitin ligase homolog gene (*RmA1H1*), that causes degradation of the AQP *PIP2;1* and inhibits its transport, exhibited significantly increased drought tolerance compared to wild-type (WT) plants [91]. Consistent with these findings the transcription level of *PIP2;1* also decreased upon dehydration stress [86,87].

In addition, *Arabidopsis* mutants overexpressing the *GoPIP1* gene from the forage crop *Galega orientalis* were more sensitive to drought and exhibited lower leaf water content as well as faster water loss through leaves and reduced rosette weight [88]. These results suggest that, especially under abiotic stress conditions, AQPs are associated with drought sensitivity. In tobacco (*Nicotiana tabacum*) the transcript levels of two AQP genes (*NtPIP1;1*, *NtPIP2;1*) were significantly decreased after drought stress [89]. At the same time the transcription level of a third AQP gene (*NtAQP1*) was significantly increased after drought stress, which might indicate different functions of AQPs during exposure to drought. Another example of a high impact AQP gene was reported in banana plants (*Musa acuminata*), where transcript levels of *MaPIP1;1* were elevated during drought stress [90]. Furthermore *MaPIP1;1* overexpressing *Arabidopsis* mutants exhibited better growth, higher survival rates and reduced water loss rates than WT plants upon drought stress [90].

In conclusion, the functions of AQPs during responses to drought stress can be characterized as diverse. AQPs are able to facilitate both sensitivity as well as tolerance to drought, probably pointing to heterogeneous functions in plants. These different functions elucidate the importance of AQPs during abiotic stress responses and illustrate their potential value for breeding drought tolerant crops [92].

### 3.3. Protection against Reactive Oxygen Species

Reactive oxygen species (ROS) in form of oxygen radicals or H_2_O_2_ are formed under drought conditions and lead to cell damage via oxidation or membrane damage. Candidate genes, which can lead to an enhanced protection against ROS during drought periods, are shown in an overview in Table 5. Detection of genetic variation in these genes within crops would allow an improved breeding for stress tolerance improving the conditions and survival of plants under suboptimal water conditions.

**Table 5:** Candidate genes for detoxification.

Gene	Gene Function	Abiotic Stress Tolerance	Reference
Aldehyde dehydrogenase family 7 member (*ALDH7*)	Oxygen radical detoxification	Extenuated oxidative stress	[51,93]
Ascorbate peroxidase (*APX*)	H_2_O_2_ metabolism	Preventing oxidative stress	[94]
Glutathione reductase (*GR*)	H_2_O_2_ metabolism	Induced by oxidative stress	[95]
Superoxide dismutase (*SOD*)	Oxygen radical detoxification	Improved drought tolerance; Induced by drought	[96–98]

The expression of aldehyde dehydrogenases (ALDHs) is upregulated under stress situations like dehydration, salinity and oxidative stress. ALDHs are able to convert highly reactive aldehydes and hence extenuate oxidative stress [99].

In a transcription profiling study of two drought tolerant Andean native potato clones (*Solanum tuberosum* subsp. *andigena*) it was reported that a member of the aldehyde dehydrogenase family 7 (ALDH7) was induced under drought stress conditions [51]. These findings are in conformity with functional analyses of an ALDH7 gene member (*GmPP55*) from soybean (*Glycine max*). Transgenic *Arabidopsis* and tobacco (*Nicotiana tabacum*) plants exhibited improved tolerance to H_2_O_2_ as well as salt and drought conditions during different developmental stages [93].

A key enzyme in the detoxification of ROS is ascorbate peroxidase (APX), which functions as a H_2_O_2_ reductant [100]. APX utilizes ascorbate, an electron donor, which enables the reduction of H_2_O_2_ to water and monodehydroascorbate under oxidative stress conditions. Monodehydroascorbate can be converted into ascorbate or dehydroascorbate. Dehydroascorbate is reduced to ascorbate by the action of dehydroascorbate reductase, which requires glutathione. This implies that the activity of ascorbate peroxidase prevents oxidative stress by H_2_O_2_ detoxification [101]. Enhanced tolerance to various stresses including salt, water and PEG-induced stress was reported from transgenic tobacco plants overproducing an *Arabidopsis* gene, encoding a cytosolic APX, in the chloroplast [94]. These results suggest that APXs are essential in plants for maintaining optimal photosynthetic rates during abiotic stress and therefore benefit survival and growth under water limiting conditions by the detoxification of ROS.

Studies of different wheat cultivars revealed another component in the detoxification process of ROS that might enable improved stress tolerance. Glutathione reductase (GR) is a part of the ascorbate-glutathione cycle; in a drought tolerant wheat cultivar, GR activity was enhanced in leaves and roots in response to osmotic stress, which might facilitate protection against ROS [95]. Hence the importance of the ascorbate-glutathione cycle as a major component is elucidated, as different components might serve as candidate genes in drought-tolerant crop breeding.

Additionally superoxide dismutases (SODs) are essential enyzmes during detoxification of ROS in plants. Depending on the metal co-factor four isoforms have been classified: copper zinc SODs (Cu/Zn-SODs), iron SODs (Fe-SODs), manganese SODs (Mn-SODs), and nickel SODs (Ni-SODs) [102]. The latter type has so far only been identified in prokaryotes. Alfalfa (*Medicago sativa*) overexpressing a SOD of the Mn-type from *Nicotiana plumbaginifolia* has been reported to exhibit enhanced vigor after water deficit as well as improved herbage yield [96]. In addition to these findings, it has been discovered that in sunflower (*Helianthus annuus*) two genes encoding Cu/Zn-SODs were significantly induced upon drought stress treatments [97]. As one of the SOD genes has also been reported to be downregulated in a drought sensitive genotype [98], a positive correlation between the expression of SODs and improved stress tolerance appears to be evident.

Conclusively, results of expression profiling as well as transgenic approaches demonstrated the importance of ROS-detoxification in plants during drought stress. Different candidate genes that are involved in the detoxification process have been reported to benefit plant growth as well as survival upon abiotic stress and therefore provide an excellent resource for improving drought tolerance in crop breeding.

## 4. Stay Green Trait

Extended periods of photosynthesis are advantageous in order to increase crop yields, whereas premature senescence due to water limitations might lead to reduced productivity [103,104,105]. Stay-green mutants that retain their greenness, which can be visually accessed or measured by photometric devices, have been connected with drought tolerance. Five types A–E have been defined for stay-green mutants [24]. The first two types A and B are so called functional stay-green mutants [106]. Type A is characterized by a delayed initiation of senescence compared to the wild type whereas type B shows a prolonged senescence. In both cases, the duration of photosynthesis is elongated. The other three types C–E belong to the non-functional stay-green mutants, also called cosmetic stay-green mutants [106]. Here the chlorophyll breakdown is impaired. In type C only the degradation of the chlorophyll is delayed, while the photosynthetic activity has already been reduced. In the type D the cells die before or in the middle of the senescence period and plants appear green. The type E is characterized by higher chlorophyll level, which needs longer time for degradation. Functional stay-green mutants have been successfully selected by conventional breeding that show higher productivity, enhanced drought tolerance and better performance under low nitrogen supply [103]. Functional stay-greens are genotypes in which the transition point between C-capture and N-remobilization is delayed, or the transition occurs on time but the subsequent yellowing and N-remobilization run slowly [24,105].

Ethylene plays a major role in leaf senescence. The dominant ethylene-insensitive mutant of *ETR1* (*etr-1*) leads to a stay-green phenotype [107], as well as a mutant of *EIN2*, a positive regulator of the ethylene signaling pathway (*ore3*, also called *oresara3*) [108]. In *Arabidopsis*, stay-green mutants of the *ACS* gene family showed reduced ethylene production as the conversion of SAM to ACC represents the rate-limiting step. Apart from ethylene, ABA promotes leaf senescence. *SAG113* encodes a member of the protein phosphatase 2C family, which is part of the ABA signaling pathway. Mutants of *SAG113* (*sag113*) show reduced ABA sensitivity. This stay-green phenotype is probably due to the ABA-mediated leaf senescence that is induced by loss of turgor [109]. All these mutants are examples for functional stay-green mutants. Furthermore, overexpression of master regulators of stress responses, C-repeat binding factor 2 (CBF2)/DREB1C and CBF3/DREB1A, results in a stay-green phenotype with enhanced stress resistance [110].

Rolando *et al.* [111] investigated the chlorophyll content and its degradation in three potato varieties under limited irrigation. Lower chlorophyll degradation rates were observed in the genotypes Sarnav and Unica that yielded higher final tuber biomass. The short-term increase of chlorophyll after drought induction was regarded as the consequence of the halted leaf growth representing the conservative survival strategy, while the riskier genotypes respond with less water-use efficiency strategy [111]. This strategy resulted in reduced yield losses under moderate stress conditions. For cassava (*Manihot esculenta* Crantz), a relatively high genetic correlation was observed between leaf retention and root yield [112]. Cassava clones with leaf retention produced more total fresh biomass and 33% more root dry matter than plants without the trait. Increased root yields under well-watered and water-limited conditions were associated with total biomass production and increased harvest index [112].

In cereals, major differences occur between the crop species with regard to the source-sink relations during the grain-filling period [103]. In maize, grain yield appears to be dependent on maintenance of high activity of the source, *i.e.*, on keeping the photosynthetic activity in the leaves up [113], whereas this has only a slight effect in wheat. In maize, inbred lines and hybrids could be identified that show delayed senescence in combination with higher grain yield [114,115,116]. The subtropical white dent maize lines CML444 and SC-Malawi also displayed higher yields under low water regime [117]. In sorghum, stay-green hybrids produced 47% more biomass under terminal drought [118,119,120]. A new wheat stay-green mutant *tasg1* showed an enhanced antioxidative capacity that leads to a delayed senescence and better drought resistance [121]. The efficient removal of ROS by the ascorbate-glutathione cycle under limited water conditions might be the key factor for the observed enhanced stress tolerance. In conclusion, in cereals the stay-green trait seems to be favorable in maize and sorghum, but not that much in wheat and barley [103].

The best option for breeding using stay-green mutants with regard to high yields under drought as well as well-water conditions would be to look for functional stay-green mutants that demonstrate normal onset of senescence combined with slow kinetics of senescence or delayed onset of senescence, but normal kinetics of senescence. This can be coupled with, e.g. early flowering as stay-green stands for a prolonged time of green leaf area after anthesis. Drought tolerance is an important breeding goal that can gain from screening for stay-green mutants.

## 5. Conclusions

Research on abiotic stress caused by limited water supply has unraveled complex networks and cross talks between plant hormones and metabolic pathways. The strategies that have evolutionarily evolved for the survival of plants [122] need now to be employed to guarantee high crop yields and yield stability under drought conditions [14]. Some strategies might be combined with negative agricultural traits or might require too much energy and resources during the development of the plants so that drought tolerance is linked with reduced yields. Tolerance might be only favorable under conditions of limited water supply [123], while under well-water conditions drought tolerant crops might show lower yields. Recent studies on drought tolerance in potato have shown that drought tolerance may be linked to yield penalty [124]. Under field conditions water is at least for some time freely available and additional genotype x environment interactions also affect the yield giving a different picture than under controlled conditions [10,125].

Transgenic approaches have been useful in proof-of-concept studies to understand the molecular mechanisms underlying drought tolerance. However, the majority of transgenic approaches have not been successful in regard to obtaining high yielding drought tolerant crops because strong constitutively expressed promoters instead of drought-regulated or tissue-specific ones have been used to overexpress candidate genes for drought tolerance [10,126]. Unwanted pleiotropic effects as well as yield penalties for employing metabolically cost-intensive pathways not required under well-watered conditions were observed [127]. In addition, in most cases survival (and/or recovery) of drought in model plants like *Arabidopsis* was investigated, not the effect on yield and productivity in crops [128]. Different genetic engineering strategies for abiotic stress and target genes are outlined by Cabello *et al.* [127]. Transgenic approaches using drought inducible promoters, tissue-specific expression [126] or synthetic biology approaches [127] might be successful in the future in improving drought tolerance in crops.

Breeding for drought tolerance is challenging, but absolutely essential under the expected climate changes that might lead to more frequent periods of low water supply, especially in subtropical regions. The extensive compilation of plant drought stress genes and their homologs in nine species in the new database (DB), DroughtDB represents a helpful tool to understand and to develop concepts for drought tolerant crops [129]. As a next step, mining the natural genetic variation in candidate genes for drought tolerance in germplasm material or induced genetic variation in ethyl methansulfonate (EMS) mutagenized populations by using next generation sequencing technologies [130] or ecotilling [131] would be desirable to associate changes on the molecular level with drought relevant traits in order to develop markers that facilitate selection for drought tolerance in breeding programs. On the other hand, the whole reprogramming plants undergo under drought and rehydration can be analyzed using omics technologies [9,132]. On the whole genome level genomic selection might offer a possibility to breed efficiently for drought tolerance by selecting favorable individuals on the basis of the genomic estimated breeding values (GEBVs) [133]. Modelling the behavior of a crop under drought conditions will also help to improve crop productivity under water saving irrigation systems [6,7,134].

Even though an enormous amount of knowledge with regard to drought tolerance has been gained in the recent years, we are still far from understanding all underlying mechanisms and the signaling pathways involved. A huge effort is now required to transfer this knowledge to the various crops. Field trials estimating yields under well-watered and drought conditions have to be performed to successfully breed for drought tolerance in crops.
